# DNA Methylation Analysis of Chromosome 21 Gene Promoters at Single Base Pair and Single Allele Resolution

**DOI:** 10.1371/journal.pgen.1000438

**Published:** 2009-03-27

**Authors:** Yingying Zhang, Christian Rohde, Sascha Tierling, Tomasz P. Jurkowski, Christoph Bock, Diana Santacruz, Sergey Ragozin, Richard Reinhardt, Marco Groth, Jörn Walter, Albert Jeltsch

**Affiliations:** 1 School of Engineering and Science, Jacobs University Bremen, Bremen, Germany; 2 Institut für Genetik, FB Biowissenschaften, Universität des Saarlandes, Saarbrücken, Germany; 3 Max-Planck-Institut für Informatik, Saarbrücken, Germany; 4 Max Planck Institute for Molecular Genetics, Berlin-Dahlem, Germany; 5 Leibniz-Institute for Age Research—Fritz-Lipmann-Institute, Jena, Germany; Friedrich Miescher Institute for Biomedical Research, Switzerland

## Abstract

Differential DNA methylation is an essential epigenetic signal for gene regulation, development, and disease processes. We mapped DNA methylation patterns of 190 gene promoter regions on chromosome 21 using bisulfite conversion and subclone sequencing in five human cell types. A total of 28,626 subclones were sequenced at high accuracy using (long-read) Sanger sequencing resulting in the measurement of the DNA methylation state of 580427 CpG sites. Our results show that average DNA methylation levels are distributed bimodally with enrichment of highly methylated and unmethylated sequences, both for amplicons and individual subclones, which represent single alleles from individual cells. Within CpG-rich sequences, DNA methylation was found to be anti-correlated with CpG dinucleotide density and GC content, and methylated CpGs are more likely to be flanked by AT-rich sequences. We observed over-representation of CpG sites in distances of 9, 18, and 27 bps in highly methylated amplicons. However, DNA sequence alone is not sufficient to predict an amplicon's DNA methylation status, since 43% of all amplicons are differentially methylated between the cell types studied here. DNA methylation in promoter regions is strongly correlated with the absence of gene expression and low levels of activating epigenetic marks like H3K4 methylation and H3K9 and K14 acetylation. Utilizing the single base pair and single allele resolution of our data, we found that i) amplicons from different parts of a CpG island frequently differ in their DNA methylation level, ii) methylation levels of individual cells in one tissue are very similar, and iii) methylation patterns follow a relaxed site-specific distribution. Furthermore, iv) we identified three cases of allele-specific DNA methylation on chromosome 21. Our data shed new light on the nature of methylation patterns in human cells, the sequence dependence of DNA methylation, and its function as epigenetic signal in gene regulation. Further, we illustrate genotype–epigenotype interactions by showing novel examples of allele-specific methylation.

## Author Summary

Epigenetics is defined as the inheritance of changes in gene function without changing the DNA sequence. Epigenetic signals comprise methylation of cytosine bases of the DNA and chemical modifications of the histone proteins. DNA methylation plays important roles in development and disease processes. To investigate the biological role of DNA methylation, we analyzed DNA methylation patterns of 190 gene promoter regions on chromosome 21 in five human cell types. Our results show that average DNA methylation levels are distributed bimodally with enrichment of highly methylated and unmethylated sequences, indicating that DNA methylation acts in a switch-like manner. Consistent with the well-established role of DNA methylation in gene silencing, we found DNA methylation in promoter regions strongly correlated with absence of gene expression and low levels of additional activating epigenetic marks. Although methylation levels of individual cells in one tissue are very similar, we observed differences in DNA methylation when comparing different cell types in 43% of all regions analyzed. This finding is in agreement with a role of DNA methylation in cellular development. We identified three cases of genes that are differentially methylated in both alleles that illustrate the tight interplay of genetic and epigenetic processes.

## Introduction

After deciphering the sequence of the human genome, the study of epigenetic processes which initiate and maintain heritable patterns of gene expression and gene function without changing the DNA sequence, has moved into the center of research [1]. The epigenome comprises different modifications of histone proteins including acetylation, ubiquitination, phosphorylation and methylation working in concert with methylation of the DNA [2],[3]. In mammals, DNA methylation predominantly occurs at CpG dinucleotides, the majority of which are methylated under normal cell conditions [4]. CpG sites are underrepresented in the human genome but cluster in CpG-islands which overlap with the annotated transcriptional start sites (TSS) of about 70% of all human genes [5] and mostly are unmethylated in normal differentiated cells [6]. DNA methylation has been shown to play important roles in the regulation of gene expression, development, genomic imprinting, X chromosome inactivation, and genome stability [7]–[9]. Erroneous DNA methylation contributes to the development of human cancer and multifactorial diseases [10]–[12].

Various high-throughput technologies for the analysis of DNA methylation in human genomes have been developed recently [13],[14]. In principle, these technologies are based on three approaches to discriminate the methylated and unmethylated cytosines in CpG sites. 1) Digestion of genomic DNA with methylation sensitive restriction enzymes to discriminate and/or enrich methylated and unmethylated DNA and employ two-dimensional electrophoresis [15], PCR [16], microarray [17] or paired-end sequencing [18] for analysis. These methods only provide methylation data related to the restriction enzyme recognition sites. 2) Enrichment of methylated or unmethylated fractions of genomic DNA with antibodies against methylated cytosine, methyl-CpG binding domains or other protein domains and readout by microarray or DNA sequencing [19]–[23]. The resolution of this approach is limited by the fragment size. 3) Bisulfite conversion of DNA leading to the selective deamination of cytosine but not 5-methyl cytosine [24],[25] and the sequencing of subsequently generated PCR products either directly [26] or after subcloning as done here. Next generation ultra-deep sequencing methods were recently used for the analysis of the bisulfite converted genomic DNA from Arabidopsis [27],[28], as well as for analysis of bisulfite converted DNA enriched for CpG island sequences in mouse [29]. The suitability of these methods for establishing reference maps of DNA methylation has been evaluated recently in silico [30].

The sequencing of subcloned single DNA molecules, as carried out in this study, provides the most reliable and detailed information of the methylation pattern for every single CpG site in a relatively long region of about 300 to 500 base pairs (bps), when analyzed by conventional Sanger sequencing. Furthermore, it provides qualitative and quantitative information of allele-specificity of DNA methylation. Drawbacks of this method are the relatively high costs for conventional Sanger sequencing and the time-consuming need to establish suitable primers for each amplicon of interest. Therefore, we focused our work on chromosome 21, which is the smallest human autosome. It is of special biomedical relevance due to its association with genetic diseases including trisomic 21 causing Down syndrome, which is the most common genetic cause of reduced cognitive abilities.

## Results

### Overview of the Results

We aimed to establish a comprehensive map of DNA methylation at promoter regions on chromosome 21. All protein-coding genes on chromosome 21 annotated in Ensembl, UCSC and RefSeq gene were investigated in a window from 2000 bps upstream to 500 bps downstream of the annotated transcriptional start site for CpG density and GC content. For this study, we selected genes which show an enriched CpG density in their promoter region. This includes genes which contain a CpG island in their promoter as defined by the widely used Takai/Jones criteria [31] and also genes with weaker CpG islands [20],[32],[33]. To increase coverage, we investigated more than one amplicon for a subset of genes, in particular for those with well-annotated alternative transcriptional start sites (TSS).

In total, we analyzed the DNA methylation pattern of 297 amplicons from 190 gene promoters by using bisulfite conversion, subcloning and sequencing as the major experimental method. The study was performed in five cell types, viz. human peripheral blood (mainly leukocytes), fibroblast, the human embryo kidney cell line HEK293, the human hepatocellular liver carcinoma cell line HepG2 and fibroblast cells derived from a patient with Down syndrome (trisomic 21). A statistical summary of the analysis is provided in Table 1. All methylation data obtained here are presented in an integrated web platform (http://biochem.jacobs-university.de/name21/) for visualization and download which also includes additional technical information. Furthermore, the results are provided as custom annotation tracks to be displayed in the UCSC Genome Browser [34].

**Table 1:** Summary of the Data.

genes analyzed	190
cell types analyzed	5
amplicons analyzed	297
mean amplicon length [bps]^1^	274
mean number of CpG sites per amplicon^1^	21.3
mean GC content per amplicon^1^	0.64
mean CpG density per amplicon^1^	0.74
PCR products obtained for the different amplicons in the tissues analyzed	1426
subcloned PCR products	1390
mean number of clones per PCR product	20.6
clones analyzed	28626
pyrosequencing or direct sequencing of PCR products	36
CpG sites analyzed in all cell types	29984
CpG sites cloned and sequenced	580427

1 In this calculation the primer sequences were not considered.

### Cell Type Specific Differences in DNA Methylation

**Figure 1:**
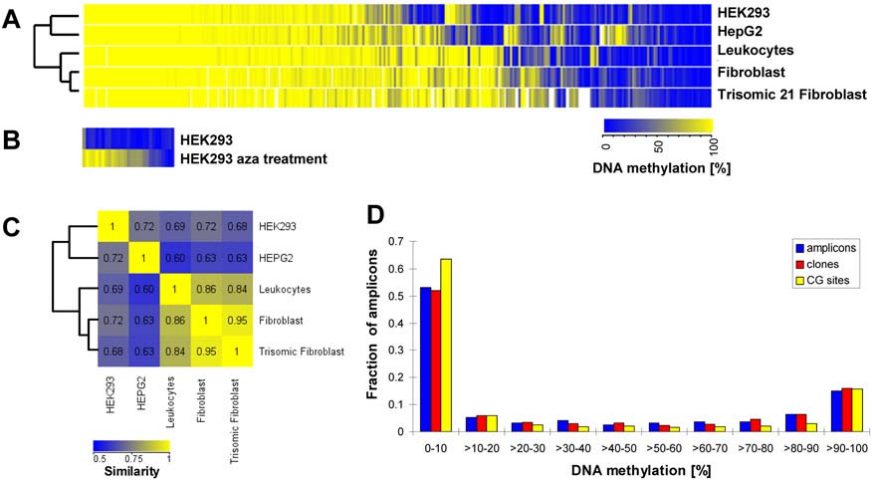
Summary of the DNA methylation data. (A) Clustered heatmap of the DNA methylation levels of all amplicons studied in HEK293, HepG2, leukocytes, fibroblast, and trisomic fibroblast cells, ordered by the average level of DNA methylation and similarity across tissues. (B) DNA methylation levels of selected highly methylated amplicons in HEK293 after treatment of the cell line with 5-azacytidine (for detail, see Text S2). (C) Tissue similarity plot comparing the methylation patterns of all five cell types studied here. The numbers represent pair wise Pearson correlation coefficients between each pair of cell types, calculated using the average DNA methylation levels for all amplicons. (D) Bimodal distribution of the DNA methylation level of all amplicons, clones, and CpG sites.

The DNA methylation levels of all amplicons in all studied cell types are shown in Figure 1A. Methylation of some of our amplicons has been studied previously. In such cases, our data in general fit well with previous results obtained for the overlapping DNA region in the same tissues (Text S1). 57% (168/297) of the amplicons show similar methylation level (methylation difference <30%) in all five cell types. The remaining amplicons are differentially methylated between two or more cell types. We clustered all five cell types according to their average DNA methylation levels for all amplicons (Figure 1C). The results show that DNA methylation levels are more similar between related cell types, e.g. between transformed cell line HEK293 and cancer cell line HepG2 and between the two types of primary cells used here, blood and fibroblasts. As seen previously, the average CpG island methylation in cultured cells or cancer cell lines is higher than in primary tissues [29],[35],[36]. We tested the effect of 5-azacytidine treatment on methylated regions in the HEK293 cells. As shown in Figure 1B and Text S2, we observed a heterogeneous response in which about 40% of the amplicons were massively demethylated but about 10% were almost completely refractory to demethyation.

As shown in Figure 1D, the methylation levels of all amplicons studied showed a bimodal distribution with an enrichment of highly methylated and unmethylated sequences, a result that as has been observed previously as well [20],[26],[29]. Most of our amplicons are situated in promoter regions and show low methylation (62% of them having methylation levels <30%). However, 25% of the amplicons are highly methylated with methylation levels >70%. The bimodal methylation level distribution was also observed at the level of the individual CpG sites and clones analyzed (Figure 1D).

### Comparison of Normal and Trisomic 21 Fibroblasts

Trisomic cells are expected to exhibit an 1.5-fold increase in gene expression levels when compared to normal cells. However, it is known that epigenetic modifications can alter such effects. For example, DNA methylation is involved in dosage compensation by X-chromosome inactivation in females [37]. To test if similar compensatory effects are mediated by methylation changes of the promoters on chromosome 21 in trisomic patients, we investigated DNA methylation patterns in trisomic fibroblasts and compared it to normal fibroblasts. In total, DNA methylation of 252 amplicons (corresponding to 169 genes) can be compared between trisomic and normal fibroblast cells. The results indicate that only a small number of amplicons (7 out of 252) are differentially methylated with a methylation difference >30% (in fact, trisomic fibroblast and normal fibroblast were most similar among all pairs of cell types studied here). Therefore, DNA methylation does not appear to be a general mechanism of global gene dosage correction in cells trisomic for chromosome 21. The data from trisomic fibroblast cells were not included in the following analyses in order to prevent statistical overweighting of fibroblast methylation data.

### Analysis of the Stability of DNA Methylation Levels

**Figure 2:**
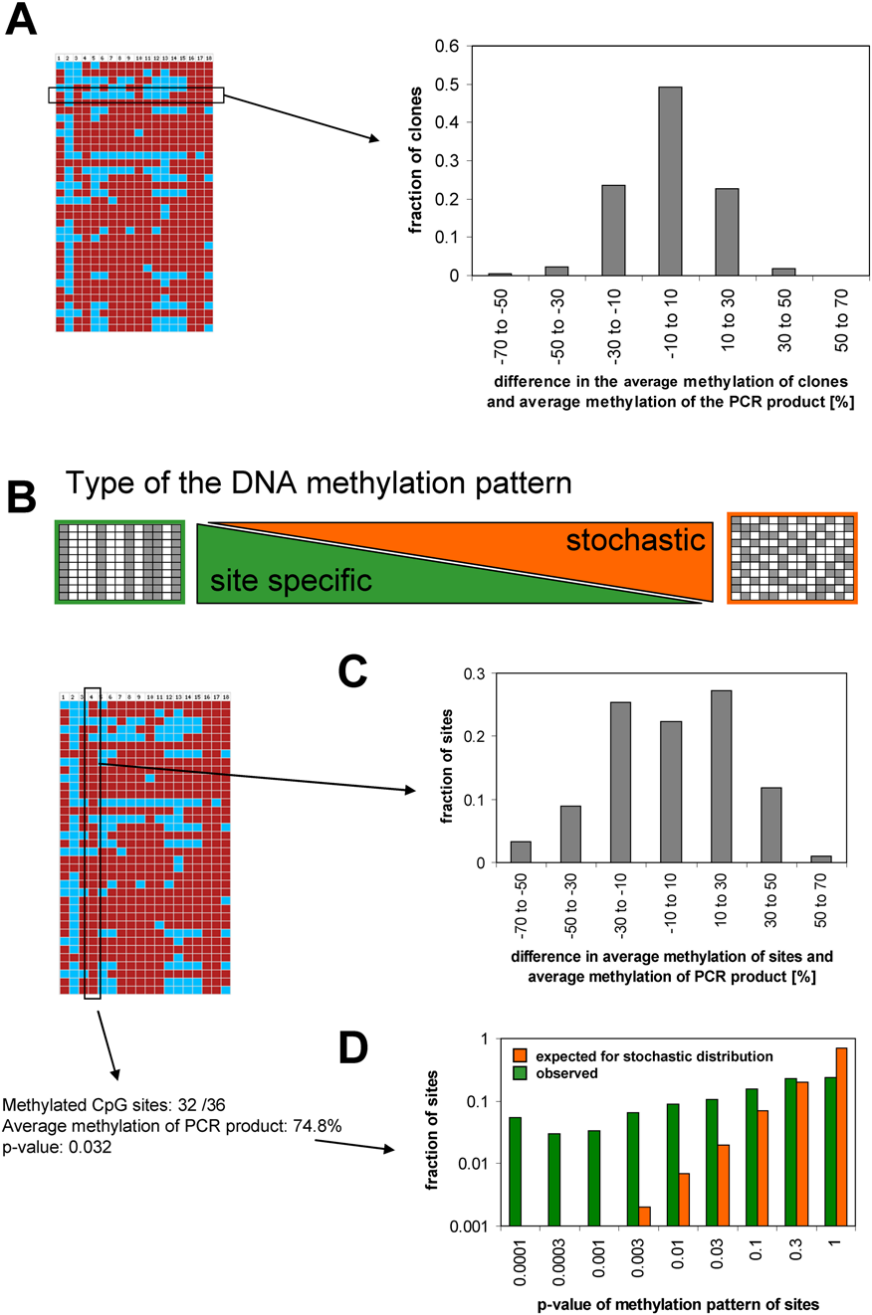
Analysis of DNA methylation patterns of amplicons. (A) Average methylation levels of clones were extracted and compared with the average methylation level of the corresponding PCR product. (B) Extreme types of possible patterns of DNA methylation being either distributed in a site-specific manner or stochastically. (C) Average methylation levels of all CpG sites from all PCR products were compared with the average methylation level of the respective PCR products, indicating that methylation levels of sites are not stochastically distributed. (D) The methylation patterns of all CpG sites were extracted and for each site the probability of the observed pattern calculated assuming a model of stochastic distribution. The distribution was plotted indicating a significant enrichment of sites with unexpected methylation pattern.

Using our high-quality set of DNA methylation profiles with single base pair and single allele resolution, we addressed the question of the stability of natural DNA methylation patterns. To this end, a subset of data was extracted only containing the PCR products with unimodal distribution of methylation levels among the clones and average methylation levels between 20 and 80%. Then, we compared the methylation levels of all clones from each PCR product in the set with the average methylation level of the respective PCR product (Figure 2A). Our data show that the methylation levels of clones from each particular PCR product were very similar to the average of the respective PCR product indicating that the methylation levels of individual cells from one tissue are similar.

### Analysis of the Distribution of DNA Methylation

We then examined the type of the DNA methylation pattern. There were two extreme possibilities, with the methylation either distributed in a site specific pattern (meaning that each site is either fully methylated or unmethylated) or in a stochastic pattern which would predict that the average methylation of each site equals the average methylation of the PCR product (Figure 2B). A stochastic pattern would only preserve the average methylation of clones but not the sites of methylation. Visual inspection of the data ruled out a strict site specific pattern. To determine if the methylation is stochastically distributed or if there are particular preferences to methylate some sites, we extracted the average methylation levels of all CpG sites of PCR products from the unimodal set and compared to the average methylation level of the respective PCR products. As shown in Figure 2C, the results significantly differ from what would be expected by a stochastic distribution, because the average methylation levels of sites cluster at levels higher and lower than the average methylation level of the corresponding PCR product. To further examine the statistical significance of this finding, the p-value of the methylation patters of each site was calculated by exact binominal test assuming a stochastic methylation pattern. As shown in Figure 2D, the fraction of sites with small p-values was much larger than statistically expected. We conclude that the methylation level of CpG sites is not strictly site specific, but there are significant differences in the methylation of individual sites, which cannot be explained by statistical fluctuation – we call it a relaxed site specific pattern.

### Differential DNA Methylation within Different Parts of Single CpG Islands

**Figure 3:**
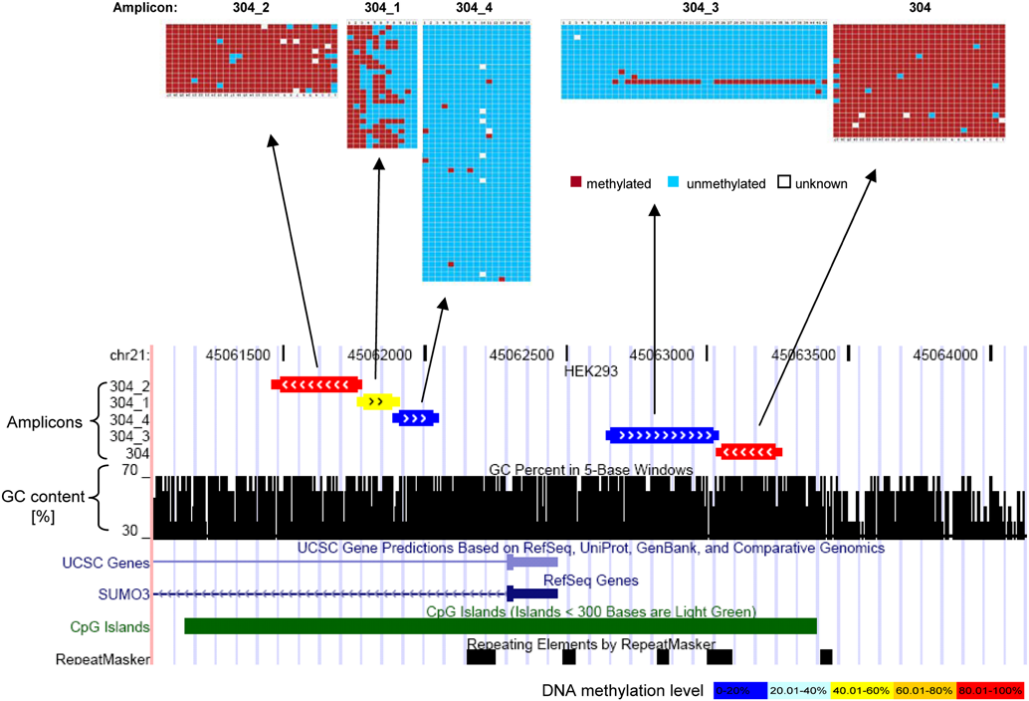
Example of amplicons located on the same CpG island but showing different DNA methylation levels and decreasing methylation when approaching the TSS of the gene. The figure shows the position of these amplicons in the UCSC genome browser in the lower part and it schematically displays the methylation pattern of five amplicons determined in HEK293 in the upper part. Each row corresponds to one sequenced clone of the bisulfite PCR product. Each column corresponds to one CpG site. The color code indicates the methylation state of the individual CpG site in each clone.

We often observed that amplicons next to each other in the same CpG island had different methylation states (see Figure 3 for an example). In our data set there are 164 examples, where more than one amplicon located on one CpG island was studied in one of the non-trisomic cell types. In 35 of these cases (21%), the amplicons were differentially methylated (with a methylation difference >30%). Differential methylation within one CpG island happened more often in HEK293 (13 out of 41, 32%) and HepG2 cells (11 out of 41, 27%), than in fibroblast cells (6 out of 40, 15%) and leukocytes (5 out of 42, 12%). It was reported that there is an unmethylated core region surrounding TSS within 1 kb of genes [26]. We checked if the amplicons on the same CpG island also show such tendency. Among the 35 cases with methylation difference on the same CpG island, 11 were not informative, because there are either two TSSs for one gene or different TSSs for two genes annotated. Hence it was not possible to determine the distance between TSS and amplicon. In 20 out of the remaining 24 cases (83%), we observed the methylation of amplicons gradually decreased when approaching the TSS of the respective gene both from upstream and downstream. An example for this is shown in Figure 3.

### Correlation of DNA Methylation with DNA Sequence

**Figure 4:**
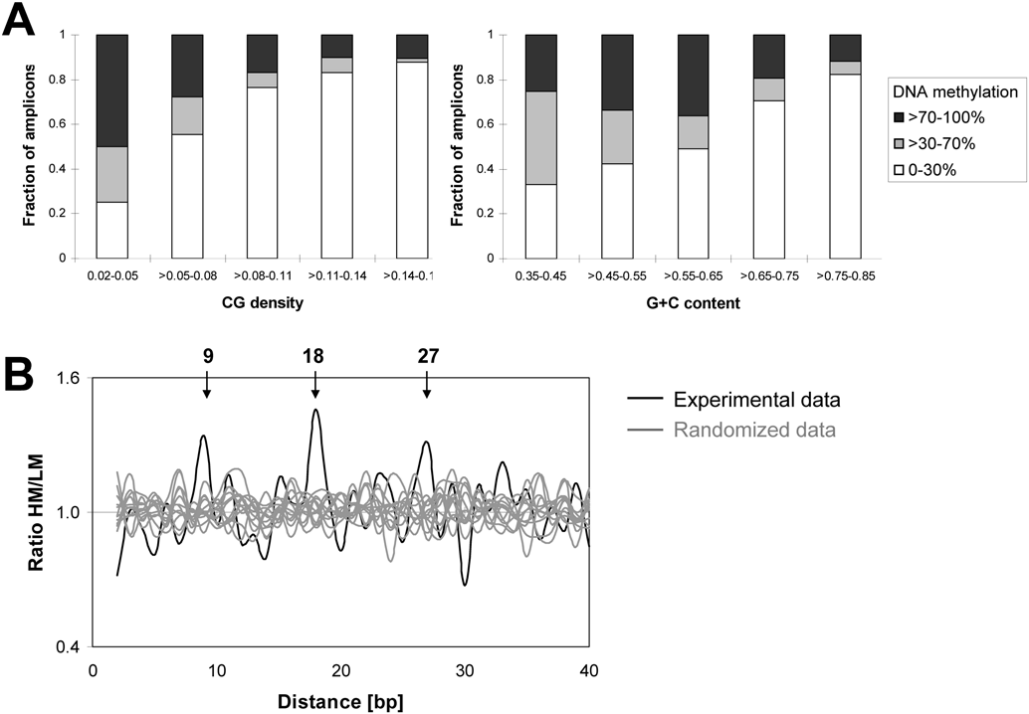
Correlation of DNA methylation and DNA sequence properties. (A) Association of DNA methylation with low CpG density and GC content. (B) Overrepresentation of distances between CpG sites in the sequences of highly methylated DNA as compared to low methylated. The occurrences of all distances of CpG sites were counted in highly methylated (HM) amplicons and low methylated (LM) amplicons. Then, the ratio of both numbers (HM/LM) was plotted versus the distance in bps (shown in black). The same calculation was performed 12 times with randomized methylation level of all amplicons (shown in light gray). The simulations were used to derive the *p*-value of the overrepresentation of the peaks at 9 bps (*p*-value <2.0×10^−5^), 18 bps (*p*-value <1.5×10^−8^) and 27 bps (*p*-value <1.1×10^−4^).

The GC content and CpG density are two critical properties for the biological effects of CpG islands. We calculated the GC content and CpG density of all studied amplicons and compared with DNA methylation. As shown in Figure 4A, the amplicons with high GC content and CpG density tend to be low methylated while those with low GC content and CpG density tend to be highly methylated, which is consistent with previous results [20],[26],[29].

The correlation between periodic distribution of CpG and DNA methylation has been reported recently [38]. We studied the distribution of CpG pairwise distances in all amplicons in our dataset. We observed three significantly overrepresented CpG distances of 9, 18, and 27 bps in highly methylated (>70%) amplicons, when comparing with low methylated (<30%) amplicons (Figure 4B). Simulations using data sets in which the observed DNA methylation levels were randomly connected to the amplicon sequences, indicated that these overrepresentations are highly significant with p-values <1.1×10^−4^ (Figure 4B). The same result was also obtained after separating amplicons for methylation <50% and >50% (data not shown). The overrepresentation of pairs of CpG sites in distances of 9 bps and multiples of this in the sequence of highly methylated DNA could be correlated to the preference of the DNMT3a DNA methyltransferase for methylation of CpG pairs in that particular distance [38],[39].

**Figure 5:**
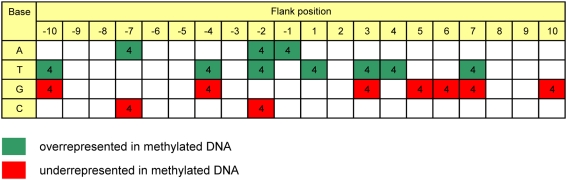
Over- and under-representation of bases in the flanks of methylated CpG sites using different threshold of DNA methylation (≥90%, ≥80%, ≥70%, or ≥60%). The numbers indicate how often a significant *p*-value was observed in the four data sets. Here only data are shown which were significantly biased with all four tested thresholds. Green and red colors indicate that the base is significantly over- or under-represented, respectively, in the flanks of methylated CpG sites. As an example, the *p*-values observed for overrepresentation of flanking nucleotides at methylation levels ≥90% are shown in Text S3.

With our large dataset of methylation states of CpG sites, we studied the effect of flanking sequence on DNA methylation. The flanking sequences (20 bases) of each methylated CpG site were collected using different methylation thresholds (Figure 5 and Text S3) and the occurrences of all four bases at each position were compared with the flanking sequences of all CpG sites used as reference. The results revealed that several bases at different positions were significantly over- or underrepresented in the flanking sequences of methylated CpG sites with p-values <1.25×10^−4^ (Text S3). These differences were reproducible when using different thresholds of methylation level (Text S3) and using different combinations of data from only two or three cell types (data not shown). No statistically significant fluctuations were detected when using randomized methylation data for the same analysis. From the overall point of view, A/T flanks correlate with DNA methylation while G/C flanks are correlated with the absence of DNA methylation. This result reflects the previous observation that *bona fide* CpG islands with high GC content and CpG density tend to be unmethylated while non-CpG islands tend to be methylated (see above). However, it is noteworthy that this tendency was not equally observed at all flanking positions.

### Inverse Correlation of DNA Methylation with Gene Expression

**Figure 6:**
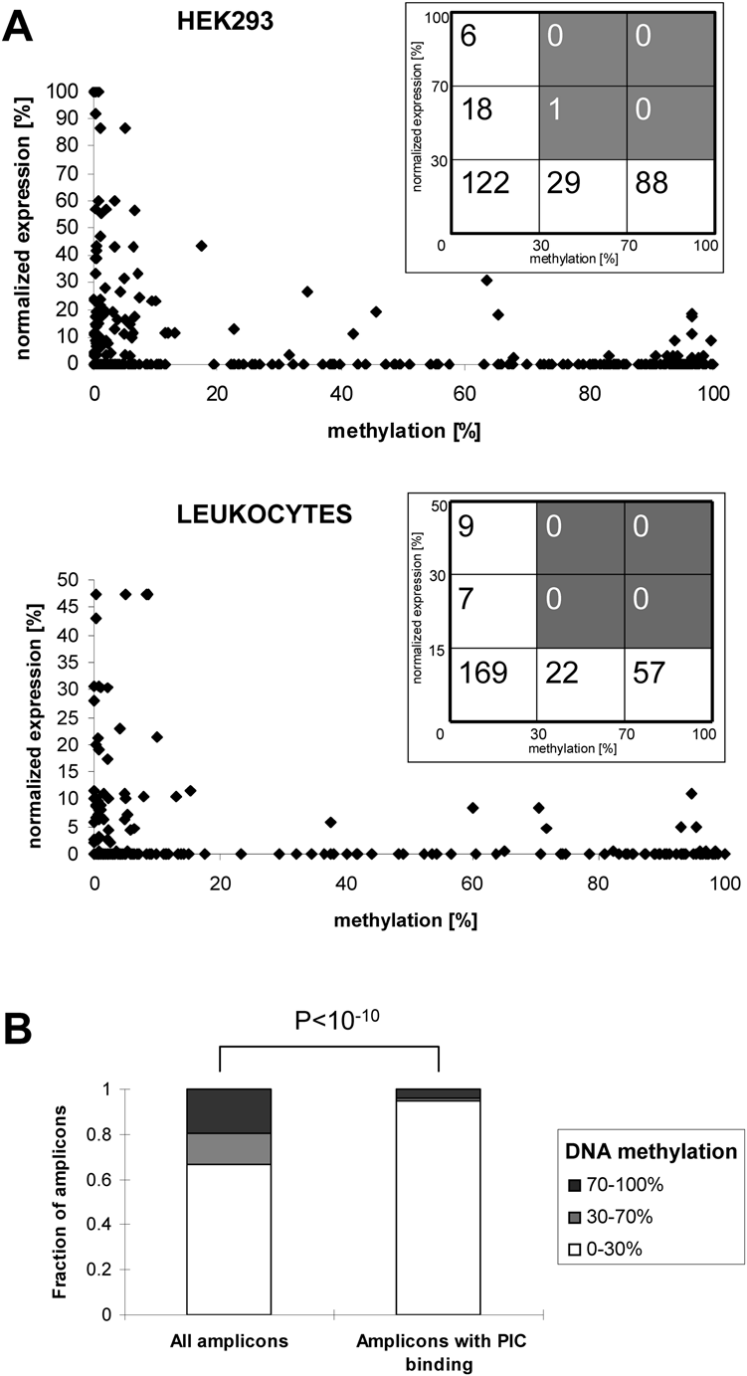
Correlation of DNA methylation and gene expression. (A) Inverse correlation of DNA methylation and gene expression. DNA methylation data obtained in HEK293 and leukocytes were plotted against the normalized SAGE expression data in the respective cell type. (B) Inverse correlation of DNA methylation and PIC binding. The *p*-value for the enrichment of unmethylated genes in the amplicons with PIC binding was calculated by exact binomial test.

DNA methylation is known to lead to gene silencing [8]. However, so far this observation was mainly based on experiments with individual genes. We compared our gene promoter methylation data with gene expression data extracted from a serial analysis of gene expression (SAGE) database [40]. Matching data sets were available for HEK293 and leukocytes. In both cell types, high methylation of amplicons is correlated with low gene expression, and high gene expression is correlated with low methylation (Figure 6A), demonstrating that DNA methylation and gene expression are inversely correlated. Some methylated amplicons from expressed genes have been excluded from this analysis, because there was more than one amplicon analyzed for that particular gene and at least one was found to be unmethylated or because there were alternative start sites annotated for the gene (Text S4).

The distribution of RNA polymerase II pre-initiation complex (PIC) is another indicator of gene expression. Kim et al (2005) mapped the PIC binding sites across the genome by immunoprecipitation of TFIID-bound DNA from primary fibroblast cells [41]. We extracted all 92 PIC binding positions that are within 2.5 kb of the annotated TSS of genes on chromosome 21. These PIC binding sites are related with 69 gene promoters, out of which 67 genes (with 93 amplicons) are with DNA methylation data. 95% of these gene promoters (88 out of 93) exhibit low levels of DNA methylation (<30%) in fibroblast. Comparing with the proportion of low methylated genes in the whole dataset of fibroblast (67%), the absence of DNA methylation is highly significant in the genes occupied with PIC (with p-values <10^−10^ according to exact binominal test) (Figure 6B). The absence of methylated genes in the PIC occupancy data set is consistent with the inhibitory role of DNA methylation on gene expression.

### Correlation of DNA Methylation with Other Chromatin Marks

**Figure 7:**
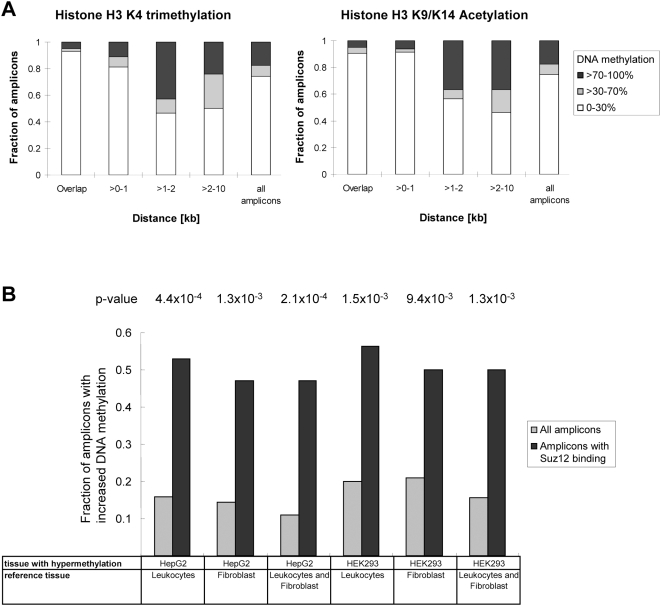
Correlation of DNA methylation and histone modification. (A) Association of low DNA methylation levels with high levels of histone H3K4me3 and histone H3K9ac/K14ac. The distances between literature-derived H3K4me3 and H3K9ac/K14ac sites in HepG2 cells and amplicons studied in this project were calculated. The fractions of amplicons with different methylation level were plotted for various distances. (B) Enrichment of polycomb binding sites in amplicons with increased methylation in HEK293 or HepG2 cells as compared to fibroblasts or leukocytes. The figure displays the fraction of amplicons with increased methylation (>30%) within the whole data set (light grey bars) and within Suz12 bound amplicons (black bars) (which is indicative of polycomb binding). *p*-values are given for each comparison.

Beside DNA methylation, histone modification is another important mechanism of epigenetic regulation of gene expression. Therefore, we assessed the correlation of DNA methylation and the following histone modifications: (i) histone H3 lysine 4 trimethylation (H3K4me3) and (ii) histone H3 lysine 9 and 14 acetylation (H3K9ac/H3K14ac), all of which are known as activating marks [3]. Their distributions were mapped across the nonrepetitive portions of chromosome 21 and 22 in HepG2 cells [42], which allowed us to correlate them with the HepG2 DNA methylation data. The distances between the regions with DNA methylation data and histone modifications were calculated up to 10 kb. The results indicate a strong correlation between the absence of DNA methylation and the presence of H3K4me3 and H3K9ac/K14ac, up to distances of 1 kb (Figure 7A).

Polycomb-mediated histone H3K27 methylation was found to pre-mark genes for de novo methylation in cancer [43],[44]. Suz12 is one core subunit of PRC2 and is essential for its activity. A genome-wide mapping of Suz12 binding sites in human embryonic stem (ES) cells revealed that Suz12 is enriched at a special set of developmental genes which are generally repressed to maintain pluripotency of ES cells and preferentially activated during ES cell differentiation [45]. It was also demonstrated that Suz12 co-occurred with histone H3K27 trimethylation at most genes [45]. We extracted the published Suz12 binding data on chromosome 21 and correlated them with our DNA methylation data. 17 amplicons from 13 genes were exactly overlapping with Suz12 binding sites (Text S5). We observed that almost half of all amplicons with polycomb binding show increased methylation in the cancer cell line HepG2 and/or the transformed cell line HEK293 which corresponds to a high enrichment when compared to the whole dataset where only about 15% show increased methylation (Figure 7B).

### Analysis of Functional Categories of Genes

**Figure 8:**
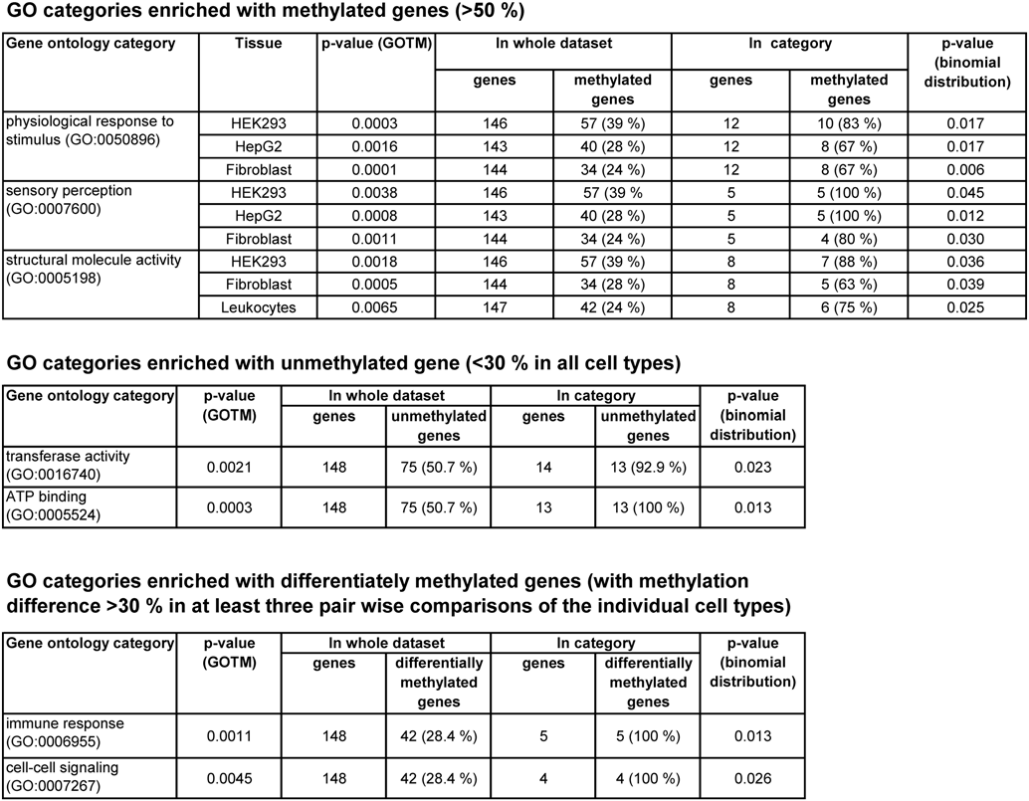
Gene ontology analysis of genes with different methylation states.

To check the biological functions associated with genes having methylated or unmethylated promoters, we analyzed the gene ontology (GO) categories of genes with methylated promoter (>50%) in at least one of the cell types, genes that were low methylated (<30%) in all studied cell types and genes with differentially methylated promoter among cell types (methylation difference >30% in at least three pair wise comparisons) by using GOTM (Gene Ontology tree machine: http://bioinfo.vanderbilt.edu/gotm/) [46]. All genes analyzed with GO annotations were used as reference list. The analysis revealed that genes with methylated or unmethylated promoters are significantly overrepresented in distinct GO categories (Figure 8). The genes with methylated promoters are overrepresented in “sensory perception” and “physiological response to stimulus” categories or they encode for structural genes like collagens while unmethylated genes are significantly overrepresented in “transferase activity” and “ATP binding” categories. Genes with differentially methylated promoters are overrepresented in “immune response” and “cell-cell signalling” categories.

### Detection of Allele-Specific DNA Methylation

**Figure 9:**
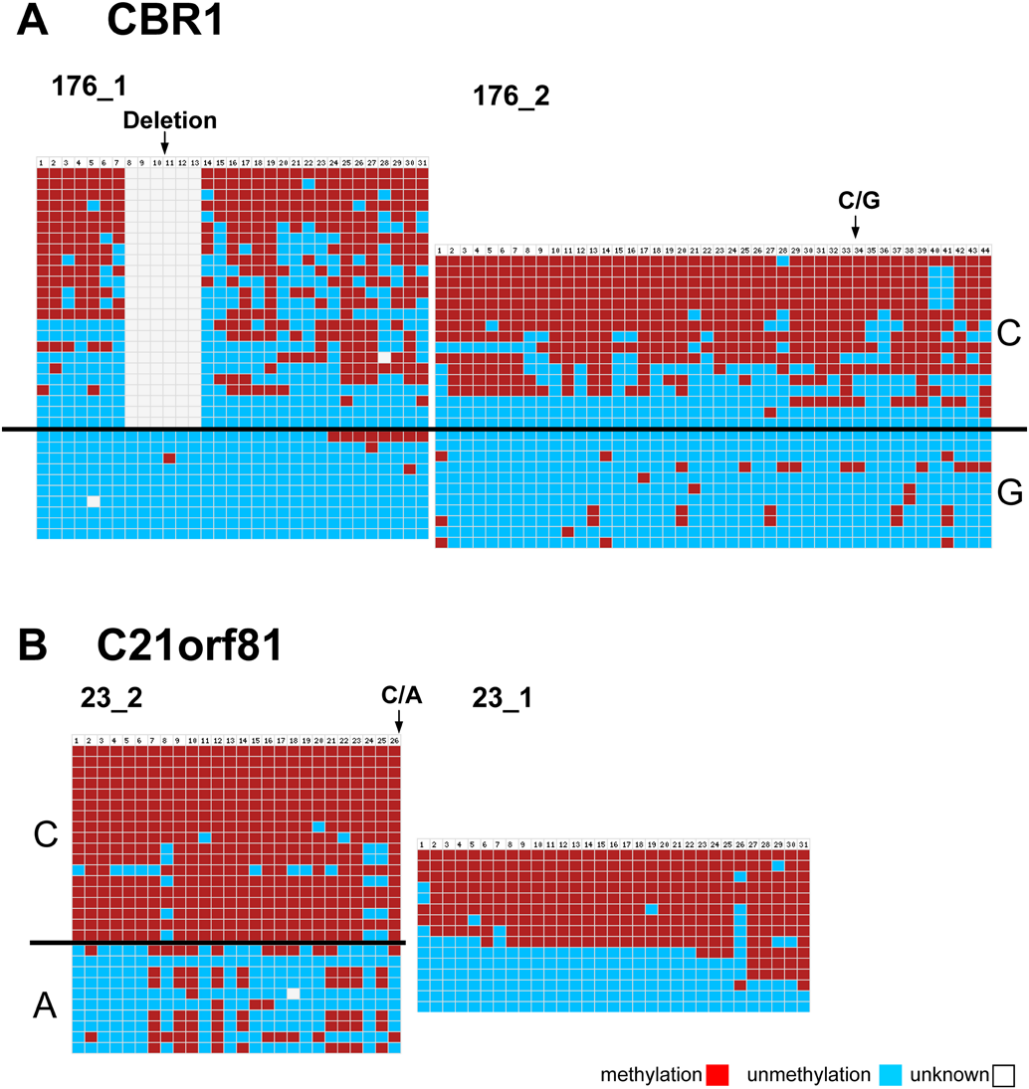
Allele-specific DNA methylation. (A) Allele-specific methylation of two amplicons (176_1 and 176_2) in the promoter of the CBR1 gene. For 176_1 clones were sorted according to the presence of a deletion (dbsnp rs41563015) in the bisulfite sequencing results. For 176_2, the clones were sorted according to the presence of C/G SNP (dbsnp rs25678). (B) Allele-specific methylation of amplicon 23_2 from gene C21orf81. A/C SNP (dbsnp rs56270809) was used to differentiate alleles. The positions of all SNP sites are indicated by arrows. Each row corresponds to one clone of bisulfite PCR products. Each column corresponds to one CpG site in the studied region. The color codes indicate the different methylation states of each CpG site in each clone. See Text S6 for details on the methylation patterns of these amplicons in other tissues analyzed.

Different methylation levels of the two alleles of one gene within one cell (allele-specific DNA methylation) has been observed in imprinting regions, where methylation of one allele occurs on a parent of origin basis [47] and in X chromosome inactivation in females [48]. There are also reports about sequence dependent allele-specific methylation in non-imprinted loci the in human genome [49]. Using our single allele resolution DNA methylation dataset, we checked for the presence of allele-specific DNA methylation on chromosome 21. To this end, the data were filtered for a biphasic distribution of DNA methylation levels. Hits were then manually inspected for the occurrence of SNPs in the sequenced region which can be used to differentiate the alleles. Our results indicated the presence of allele-specific methylation in three regions in leukocytes derived from a healthy individual (Figure 9 and Text S6).

Two amplicons (176_1 and 176_2) on a CpG island overlapping with the first exon of the CBR1 gene (carbonyl reductase 1) showed strong allele-specific methylation in leukocytes (Figure 9A). For amplicon 176_2 the C allele was highly methylated (average: 66%), while G allele was low methylated (average: 7%). For amplicon 176_1 about half of the clones showed a deletion and were partially methylated, while all clones without the deletion were unmethylated. This result suggests that the allele-specific methylation spans the whole CpG island. In other tissues, we did not observe a SNP in these amplicons. In HepG2, biphasic methylation was observed, while in HEK293 and fibroblast both amplicons were completely unmethylated (Text S6).

Amplicon 23_2 located on the first exon of gene C21orf81 showed massive allele-specific methylation in leukocytes with the A and C alleles methylated to 28% and 94% on average (Figure 9B). Amplicon 23_1 immediately adjacent to 23_2 also showed biphasic methylation in leukocytes, but did not contain an SNP. In other tissues, no allele-specific methylation was observed (Text S6). Amplicon 197_intern located in an internal region of gene DSCR3 showed ASM in leukocytes with the A allele either being methylated or unmethylated and the C allele always being unmethylated (Text S6). This result agrees with data obtained by Yamada et al (2004) who identified this region of allele-specific methylation in blood previously [16]. For this amplicon, fibroblast and trisomic fibroblast contained the SNP but were both unmethylated, HepG2 showed only the A allele and was unmethlylated and HEK293 showed biphasic methylation but only contained the C allele.

## Discussion

For many years, it was believed that CpG islands are mainly unmethylated and there exists little difference in the DNA methylation of different cell types. Here, we show for five different cell types that CpG islands frequently are methylated and that there is a substantial difference in the methylation pattern of different cell types. Based on the most commonly used criteria for defining CpG islands (GC content ≥55% and CpG observed vs. expected ≥0.65 [31]), 14% of the amplicons on CpG islands show dense methylation in different cell types. We also observe that a significant number of CpG islands exhibit substantial differences in their average DNA methylation levels in different parts. This result underscores the importance of the position where DNA methylation is studied, in order to draw valid biological conclusions. Our data confirm that high levels of DNA methylation in CpG rich promoters are strongly associated with down-regulation of gene expression. However, the inverse relation does not hold, because there are many examples of unmethylated genes that are not expressed, possibly due to lack of expression caused by other mechanisms than DNA methylation like absence of the relevant activating transcription factors.

With bisulfite subcloning and sequencing technology, we provide methylation data at single-allele resolution, which makes it possible to investigate cell-specific mosaicism and allele-specific methylation patterns. Methylation levels of different cells in the same tissue are very similar in general. We observed allele-specific methylation at two regions on chromosome 21 that were not identified before and also confirmed allele specific methylation at one region previously shown to be specifically methylated in the maternal allele [16]. Interestingly, our data show that allele-specific methylation in all three cases is not observed in all tissues analyzed. Further studies have to determine if the methylation differences were due to parent of origin dependent methylation that has been lost in some tissues or if some tissues underwent allele-specific methylation changes that were triggered by the genetic polymorphisms between the two alleles, which would provide an example of genotype-epigenotype interactions. Allele-specific methylation might contribute to allele-specific expression that is a widespread phenomenon in the human genome [50],[51].

There are interesting connections between DNA sequence and DNA methylation. We and others observed a strong anti-correlation of DNA methylation with CpG density and GC content, indicating that DNA methylation declines the more the sequence resembles a CpG island [20],[26],[29]. This trend is also reflected in our flanking sequence preferences of DNA methylation showing that highly methylated CpG sites are flanked by A/T rich sequences while unmethylated ones tend to be embedded in G/C rich sequences. The underlying mechanism of this phenomenon is not clear at present, however, the flanking sequence preferences suggest that not all neighboring base pairs are of equal importance for the DNA methylation state. One potential connection between DNA methylation and DNA sequence could be the flanking sequence preferences of the DNA methyltransferases. We observed an overrepresentation of A at −1 and T at +1 flanking position which agrees with the experimental flanking preferences of Dnmt3a and 3b [52]. This observation suggests a potential link to the sequence preference DNMT3a and 3b and confirms a similar conclusion based on a smaller data set [52] although preferences observed here at larger distances to the CpG site are slightly different, which is most likely due to the smaller amount of data used in the previous analysis.

Dnmt3a and 3L have been shown to form heterotetramers with two active sites in a distance of 8–10 bps and both Dnmt3a/3L and Dnmt3a tend to co-methylate CpG sites in that distance [38],[39]. The similar periodicity in the occurrence of CpG sites was also observed in the differentially methylated regions from 12 maternally imprinted mouse genes [38]. In the *Arabidopsis* genome, a periodicity of 10 nucleotides was found for CHH methylation [27]. Here, we observe for the first time a genome wide enrichment of pairs of CpG sites in distances of 9, 18 and 27 bps in highly methylated DNA when compared to unmethylated DNA. This effect could be due to the preferential methylation of sites in such distances. The occurrence of multiples of 9 could be related to the multimerisation and protein nucleofilament formation observed with Dnmt3a and Dnmt3a/3L [38],[39].

However, our results clearly illustrate that DNA methylation is a dynamic mark, the pattern of which cannot be explained at the level of the DNA sequence alone, because 43% of the amplicons (129 out of 297) showed significant differences in DNA methylation between at least two out of the five cell types analyzed. In extrapolation to the total number of cell types in the human body, it is likely that most of the CpG islands will show differential methylation in some cell types. DNA methylation is tightly connected to other forms of epigenetic signaling. We observed a strong anti-correlation of DNA methylation and histone H3K4me3 and H3K9ac/K14ac indicating that these activating marks and the repressive DNA methylation mark are mutually exclusive at the genomic scale. Similarly as reported previously [21],[53], we observe that methylation of polycomb marked genes tends to be increased in cancer or transformed cells suggesting that polycomb signaling is connected to DNA methylation, perhaps by recruitment of DNA methyltransferases by polycomb proteins. Our data also shed some light on the biological function of epigenetic gene regulation. Unmethylated genes are significantly overrepresented in functional categories, which suggest important metabolic functions of these unmethylated genes similarly as originally proposed for CpG island associated genes [54]. The functional categories of genes with methylated promoters suggest that DNA methylation has an important effect of on regulation of cell type specific genes determining both cellular physiology and morphology.

## Materials and Methods

### Cell Culture and Genomic DNA Extraction

Blood DNA was extracted using QiaAmp Blood DNA Mini kits (Qiagen). Human embryo kidney (HEK293) cells were grown in Dulbecco's Modified Eagle's Medium (DMEM) with 10% (v/v) fetal bovine serum (FBS) at 37°C in 5% (v/v) CO_2_. Human hepatocellular carcinoma cells HepG2 (ATCC: HB 8065) were cultured in 90% RPMI 1640 supplemented with 10% FBS at 37°C in 5% (v/v) CO_2_. Fibroblast cells (CCD-1059SK) were cultured in Eagle's Minimum Essential Medium (EMEM) supplemented with 10% FBS at 37°C in 5% (v/v) CO_2_. Trisomic 21 fibroblast cells (Coriell Cell Repositories: AG08941) were grown in EMEM with Earle's salts and non-essential amino acids supplemented with 15% FBS at 37°C in 5% (v/v) CO_2_. The genomic DNA from cells was extracted using QIAamp DNA Mini kit (Qiagen). For the demethylation treatment of HEK293 cells, 2 µM 5-azacytidine was added to the media. During three days of treatment, the media was exchanged daily and cells were harvested five days after starting the experiment.

### Bisulfite Conversion, Subcloning, and Sequencing

Bisulfite methylation analysis was performed basically as described [55]. Briefly, the CpG island searcher program (http://www.cpgislands.com) [56] or the CpGPlot program (http://www.ebi.ac.uk/Tools/emboss/cpgplot/index.html) were used to check the presence of a CpG island or CpG rich region in gene promoter. RepeatMasker software (http://www.repeatmasker.org) was used to identify the presence of repeat sequences. Bisearch (http://bisearch.enzim.hu) [57] and Methprimer (http://www.urogene.org/methprimer/index1.html) [58] programs were used to design primers. For bisulfite conversion, 200–300 ng genomic DNA were digested with *Bam*HI or *Sph*I (40 U) at 37°C overnight, converted with bisulfite as described [55] and used for PCR. The PCR products were purified by ChargeSwitch PCR Clean-Up Kit (Invitrogen) and subcloned using the StrataClone kit (Stratagene). Around 40 clones for each amplicon were picked and sequenced. Plasmid DNA of clones was isolated by an automated alkaline lyses procedure, which includes template purification by PEG-precipitation and subsequently adjustment to similar molarity. DNA sequences were determined using ABI BigDye Terminator chemistry (BigDye Terminator v3.1 K) and 3730xl ABI 96-capillary sequencer systems equipped with capillaries of 50 cm separation length. The BiQ analyzer software was used to perform quality control and to derive DNA methylation patterns from the sequencing results [59]. BDPC was used to present the methylation pattern, prepare the figures and WEB presentation and compile methylation data [60].

### Flanking Sequence Analysis

The flanking sequences (20 bases) of each methylated or unmethylated CpG site in all amplicons (from non-trisomic cell types) were extracted using different thresholds of methylation level (≥90%, ≥80%, ≥70% and ≥60%). The over- or under-representation of bases (observed/expected) in the flanks of methylated CpG sites was analyzed with Microsoft Excel BINOMDIST function. The flanking sequences of all CpG sites were used as reference. Figure 5 and Text S3 summarize the bases with significant p-value (p-value <1.25×10^−4^ corresponding to p-value <0.01 when considering Bonferroni multiple testing correction) by using four different thresholds. For an additional statistical validation, we randomized the methylation data of CpG sites 10 times and extracted the respective (randomized) flanking sequences. No significant p-value was observed for the flanking sequences from the randomized datasets. We also performed the analysis by using different combination of data from only three cell types and still observed the same results.

### Correlation of CpG Periodicity and DNA Methylation

The distance distribution of pairwise distances between CpG site and the frequency of observed/expected of all pairwise distances was determined in all amplicon sequences within distances of 2–200 bps using the DISTRIDIST program (http://biochem.jacobs-university.de/cgi/distridist.cgi). Amplicons with low GC (≤0.55) and observed/expected CpG dinucleotide content (≤0.65) were excluded from this analysis, because the poor CpG density of them could have caused a bias in the data analysis. For each distance value, the ratio of averaged observed/expected CpG pairwise distances was calculated comparing highly methylated amplicons (HM, 70%–100% methylation) with low methylated amplicons (LM, 0–30% methylation). This ratio (HM/LM) reflects the over- or underrepresentation of CpG pairwise distances in the highly methylated amplicons as compared to low methylated amplicons. Since there was no difference in results between the different cell types, data were finally averaged over all cell types. To determine the statistical significance of the data, we randomized the methylation level of all amplicons 12 times and performed the same calculation. The randomized distributions were used to derive standard deviation and mean and to calculate p-values assuming normal distribution.

### DNA Methylation and PIC Occupancy

The TFIID binding positions on chromosome 21 in IMR90 fibroblast cells were extracted from published results [41] and converted from NCBI Build 34 to NCBI Build 36. The p-value for the enrichment of unmethylated genes in the amplicons with PIC binding, comparing to the whole dataset, was calculated assuming a binomial distribution.

### Gene Functional Category Analysis

The analysis was performed using GOTM (http://bioinfo.vanderbilt.edu/gotm/), which is based on hypergeometric test to show the overrepresented gene ontology categories (p-value <0.01) [46]. 148 out of 190 genes studied on chromosome 21 were used as reference gene list for the statistical analysis. The other 42 genes are without functional annotation in GOTM, so they could not be included in the analysis. The p-value was also calculated according to BINOMDIST function on the basis of the overrepresentation of gene ontology categories in methylated, unmethylated or differentiately methylated genes when comparing to all genes, as a confirmation of the significance of results.

### Correlation of DNA Methylation and Histone Modification

The published histone H3K4me3 and H3K9ac/K14ac modification data in HepG2 cells with a p-value <10^−4^ were extracted [42] and the positions of them were converted from NCBI Build 33 to NCBI Build 36. The minimal distance was calculated between regions with DNA methylation and histone modification data. All amplicons correlated with histone H3K4me3 or H3K9ac/K14ac were used as reference to show the normal distribution of amplicons according to methylation level.

### DNA Methylation and Polycomb Protein Binding

The published SUZ12 binding data on chromosome 21 in ES cells were extracted from supplementary table S7 of Lee et al., 2006 [45]. Only amplicons which are exactly overlapping with SUZ12 binding sites were included in this analysis. The different proportions of higher methylated (methylation increase >30%) amplicons in HepG2 or HEK 293 cells, comparing to that in normal leukocytes and fibroblast cells, in whole dataset and in amplicons with SUZ12 binding were calculated respectively. The p-value was calculated based on BINOMDIST function.

### Gene Expression Data Analysis

The SAGE database (http://cgap.nci.nih.gov/SAGE/AnatomicViewer) presents ranked expression data for different cell types [40]. For each gene the relative tag occurrence as tag per 200,000 is provided. Standardization of the relative expression is the key task in this experiment, because one has to discriminate low expression caused by an intrinsically weak promoter from low expression caused by silencing of a strong promoter. Therefore, we quantified the expression potential of each promoter using the six highest expression levels of each gene. This maximal expression was then used to normalize the expression level in HEK293 and leukocytes, which resulted in a percentage of gene expression. Normalized expression data were then compared with the methylation data.

## Supporting Information

Text S1 Comparison of the NAME21 data with published DNA methylation data for Chromosome 21.
(0.05 MB DOC)

Text S2 DNA methylation changes after treatment of HEK293 cells with 5-azacytidine.
(0.17 MB DOC)

Text S3 Flanking sequence correlation to DNA methylation.
(0.06 MB DOC)

Text S4 Amplicons excluded from expression analysis.
(0.14 MB DOC)

Text S5 Methylation data of amplicons overlapping with SUZ12 binding regions.
(0.05 MB DOC)

Text S6 Allele-specific methylation.
(0.06 MB DOC)
