# Supplementary material for: DNA Methylation Analysis of Chromosome 21 Gene Promoters at Single Base Pair and Single Allele Resolution
Source: PLoS Genet. 2009 Mar 27;5(3):e1000438. doi: 10.1371/journal.pgen.1000438 (PMC2653639; doi:10.1371/journal.pgen.1000438)
Supplement: Text S1 — Comparison of the NAME21 data with published DNA methylation data for Chromosome 21. (0.05 MB DOC) [file pgen.1000438.s001.doc]

**DNA methylation analysis of chromosome 21 gene promoters at single base pair and single allele resolution**

Yingying Zhang, Christian Rohde, Sascha Tierling, Tomasz P. Jurkowski, Christoph Bock, Diana Santacruz, Sergey Ragozin, Richard Reinhardt, Marco Groth, Jörn Walter, & Albert Jeltsch

**Supplemental Text S1: Comparison of the NAME21 data with published DNA methylation data for Chromosome 21**

Yamada et al. (2004) studied DNA methylation in blood by restriction protection analysis on 149 CpG islands on chromosome 21 [16], 58 of which are overlapping with 71 of our amplicons. They reported low methylation of 48 CpG islands. These regions we found low methylated in 59 of our amplicons and one amplicon showed 50.4% methylation. Yamada et al. showed high methylation for 5 CpG islands which overlap with 6 of our amplicons that are all high methylated. In addition, they found one case of allele specific methylation which we observed as well. Four amplicons were reported as incomplete methylation. In two cases, bisulfite data were shown that fit to our results (our amplicons 320 and 193_y).

Illingworth et al. (2008) established a comprehensive genome wide set of unmethylated CpG islands using whole blood [22]. Altogether 17 regions identified by them overlap with 22 amplicons studies here. 21 of the amplicons showed very low methylation (<10%) and one amplicon (196_II) which is located at the edge of the CpG island shows moderate methylation of 32 %.
