# Supplementary material for: DNA Methylation Analysis of Chromosome 21 Gene Promoters at Single Base Pair and Single Allele Resolution
Source: PLoS Genet. 2009 Mar 27;5(3):e1000438. doi: 10.1371/journal.pgen.1000438 (PMC2653639; doi:10.1371/journal.pgen.1000438)
Supplement: Text S2 — DNA methylation changes after treatment of HEK293 cells with 5-azacytidine. (0.17 MB DOC) [file pgen.1000438.s002.doc]

**DNA methylation analysis of chromosome 21 gene promoters at single base pair and single allele resolution**

Yingying Zhang, Christian Rohde, Sascha Tierling, Tomasz P. Jurkowski, Christoph Bock, Diana Santacruz, Sergey Ragozin, Richard Reinhardt, Marco Groth, Jörn Walter, & Albert Jeltsch

**Supplemental Text S2: DNA methylation changes after treatment of HEK293 cells with 5-azacytidine.**

Analysis of DNA methylation in HEK293 cells after treatment with 2 µM 5-azacytidine for 3 days. For this analysis, 29 amplicons were selected that are highly methylated in HEK293. For each amplicon analyzed the average methylation at the respective CpG site is shown. The amplicons are sorted by increasing demethylation.

| Amplicon | Gene | DNA methylation [%] | | |
| --- | --- | --- | --- | --- |
|  |  | Aza treated cells | Untreated cells | Difference in methylation |
| 187_2 | CLDN14 | 99.6 | 83.3 | -16.3 |
| 278 | DNMT3L | 93 | 87.9 | -5.1 |
| 315 | C21orf86 | 94.7 | 89.8 | -4.9 |
| 257 | CRYAA | 85.7 | 90 | 4.3 |
| 282_B | TRPM2 | 71.4 | 82.3 | 10.9 |
| 227_new3 | FAM3B | 74.6 | 92.2 | 17.6 |
| 196_II | DSCR9 | 77.1 | 98.4 | 21.3 |
| 304 | SUMO3 | 70.5 | 98.1 | 27.6 |
| 215_1 | HMGN1 | 14.7 | 45.6 | 30.9 |
| 243_a | TFF2 | 45.2 | 86 | 40.8 |
| 193_y | DSCR6 | 53.7 | 95.6 | 41.9 |
| 130 | MRAP | 50.4 | 94.3 | 43.9 |
| 307 | ITGB2 | 25.9 | 81.8 | 55.9 |
| 261 | C21orf84 | 39.1 | 95.6 | 56.5 |
| 209 | ETS2 | 39.2 | 96.5 | 57.3 |
| 193 | DSCR6 | 39.6 | 98.6 | 59 |
| 97 | KRTAP19-1 | 37.5 | 100 | 62.5 |
| 132 | C21orf77 | 30.4 | 93.8 | 63.4 |
| 196_I_x | DSCR9 | 27.8 | 91.5 | 63.7 |
| 197_intern | DSCR3 | 18.8 | 84.1 | 65.3 |
| 265_2 | KIAA0179 | 25.2 | 95.1 | 69.9 |
| 239 | C21orf128 | 11.2 | 82.4 | 71.2 |
| 196_I_y | DSCR9 | 21.7 | 93.3 | 71.6 |
| 335 | S100B | 8.9 | 84.5 | 75.6 |
| C21orf34 | C21orf34 | 12.5 | 88.4 | 75.9 |
| 235_I | C21orf25 | 14.7 | 93.9 | 79.2 |
| 317 | C21orf111 | 0 | 89.8 | 89.8 |
| 229_2 | MX1 | 0.2 | 96.2 | 96 |
| 259_1 | SNF1LK | 1.4 | 99.8 | 98.4 |

We prepared a frequency plot of the loss of DNA methylation by 5-azacytidine treatment observed with the 29 amplicons. The data show that some amplicons showed massive demethylation while other did not change their methylation state.

The degree of demethylation of the different amplicons is not correlated with any sequence specific parameter (like GC content or CpG density, presence of repeated elements). We speculate that high methylation of some amplicons is necessary for the transformed state of the cells, such that the apparent response to 5-azacytidine treatment reflects the combined influence of the susceptibility of a DNA region to demethylation and reduced viability of the cell after demethylation of some critical DNA regions.
