# Supplementary material for: DNA Methylation Analysis of Chromosome 21 Gene Promoters at Single Base Pair and Single Allele Resolution
Source: PLoS Genet. 2009 Mar 27;5(3):e1000438. doi: 10.1371/journal.pgen.1000438 (PMC2653639; doi:10.1371/journal.pgen.1000438)
Supplement: Text S3 — Flanking sequence correlation to DNA methylation. (0.06 MB DOC) [file pgen.1000438.s003.doc]

**DNA methylation analysis of chromosome 21 gene promoters at single base pair and single allele resolution**

Yingying Zhang, Christian Rohde, Sascha Tierling, Tomasz P. Jurkowski, Christoph Bock, Diana Santacruz, Sergey Ragozin, Richard Reinhardt, Marco Groth, Jörn Walter, & Albert Jeltsch

**Supplemental Text S3:** Flanking sequence correlation to DNA methylation. The number of methylated CpG sites used for flanking sequence analysis at different thresholds. The total data set comprised 24626 CpG sites (from non-trisomic cell types).

Calculation of over- and under-represented bases in the flanks of highly methylated (≥90%) CpG sites.

The colored cells are with significant p-value (<1.25 × 10^-4^ corresponding to p-value <0.01 when considering multiple testing correction). Green and red colors indicate that the base is significantly overrepresented or underrepresented, respectively, in the flanks of methylated CpG sites.
