# Supplementary material for: DNA Methylation Analysis of Chromosome 21 Gene Promoters at Single Base Pair and Single Allele Resolution
Source: PLoS Genet. 2009 Mar 27;5(3):e1000438. doi: 10.1371/journal.pgen.1000438 (PMC2653639; doi:10.1371/journal.pgen.1000438)

**DNA methylation analysis of chromosome 21 gene promoters at single base pair and single allele resolution**

Yingying Zhang, Christian Rohde, Sascha Tierling, Tomasz P. Jurkowski, Christoph Bock, Diana Santacruz, Sergey Ragozin, Richard Reinhardt, Marco Groth, Jörn Walter, & Albert Jeltsch

**Supplemental Text S4: Amplicons excluded from expression analysis.**

**A) Expressed genes with more than one amplicon analyzed:**

**DSCR3 (expressed in HEK293)**


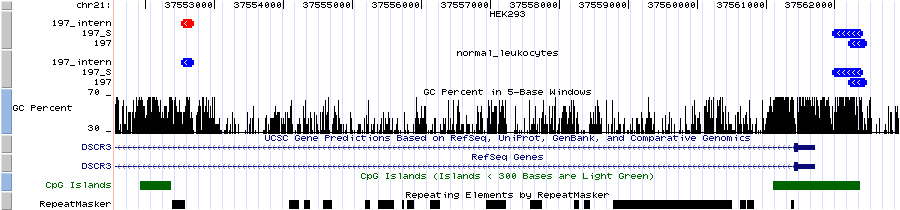


The amplicon 197_intern was excluded from this study, because it is not located in a gene promoter.

**WRB (expressed in HEK293)**


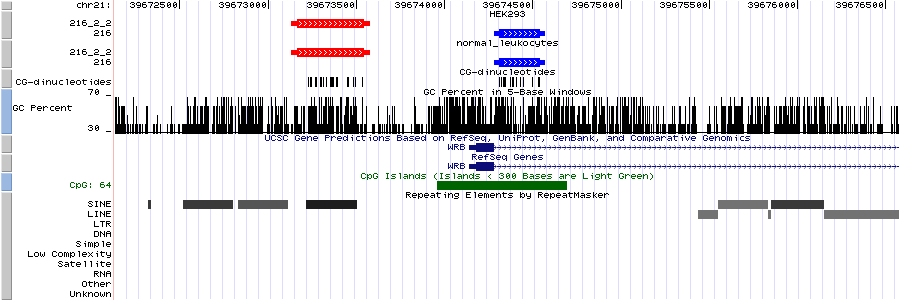


The amplicon 216_2_2 was excluded because the unmethylated amplicon 216 is on the CpG island and closer to the TSS.

**KIAA0179 (expressed in HEK293)**


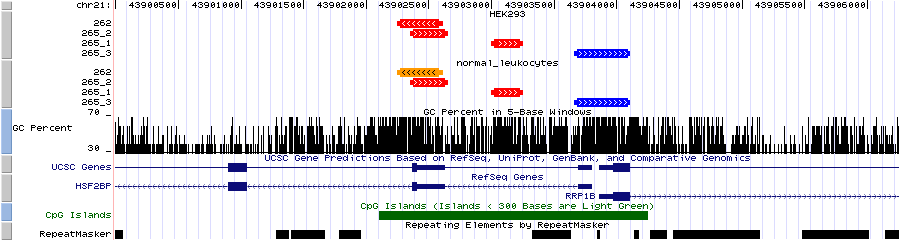


The gene KIAA0179 (RRP1B) on minus strand shares the TSS region with the gene HSF2BP on plus strand. The unmethylated amplicon 265_3 is at the best location for both genes such that 262, 265_1 and 265_2 were excluded.

**SUMO3 (expressed in HEK293)**

The promoter of this gene is shown in Fig. 3. Amplicon 304_3 is closest to the TSS and unmethylated in both tissues. All other amplicons in this region were excluded.

**C21orf56 (expressed in HEK293)**


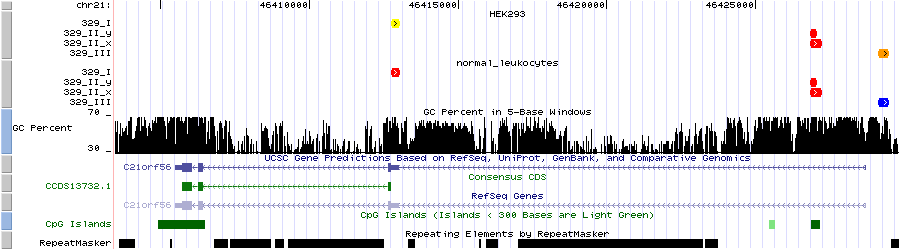


Only the amplicon 329_III was included, beause it is closest to the TSS.

**C21orf25 (expressed in HEK293)**


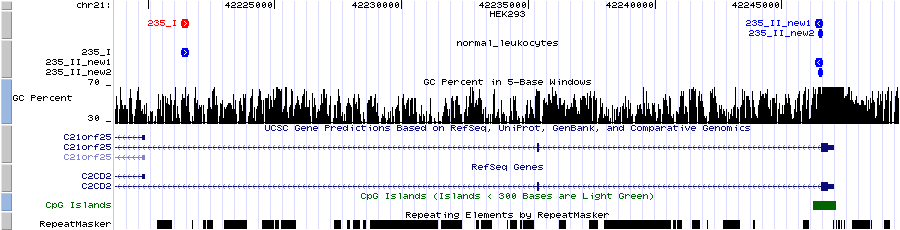


The amplicons 235_II_new1 and 235_II_new2 were used.

**C21orf124 (expressed in HEK293 and Leukocytes)**


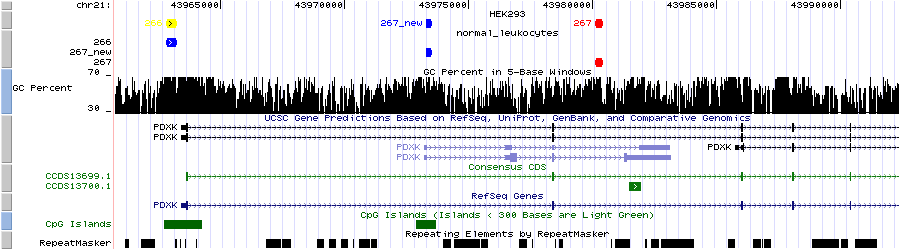


The amplicon 267 was excluded.

**B) Genes with alternative CpG poor start positions:**

**UMODL1 (expressed in HEK293 and Leukocytes)**


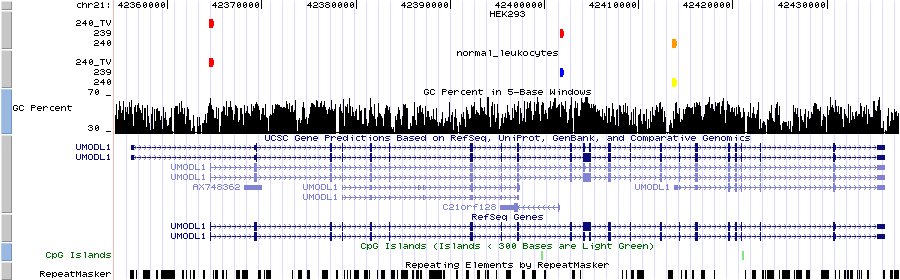


**SAMSN1 (expressed in Leukocytes)**


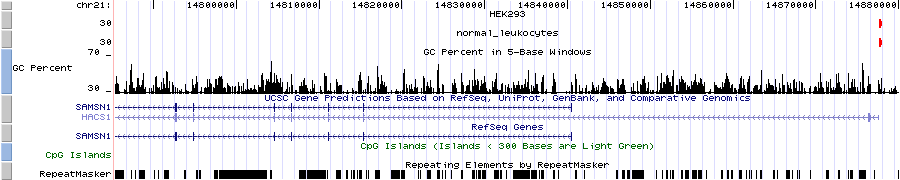

Supplement: Text S4 — Amplicons excluded from expression analysis. (0.14 MB DOC) [file pgen.1000438.s004.doc]
