# Supplementary material for: DNA Methylation Analysis of Chromosome 21 Gene Promoters at Single Base Pair and Single Allele Resolution
Source: PLoS Genet. 2009 Mar 27;5(3):e1000438. doi: 10.1371/journal.pgen.1000438 (PMC2653639; doi:10.1371/journal.pgen.1000438)
Supplement: Text S5 — Methylation data of amplicons overlapping with SUZ12 binding regions. (0.05 MB DOC) [file pgen.1000438.s005.doc]

**DNA methylation analysis of chromosome 21 gene promoters at single base pair and single allele resolution**

Yingying Zhang, Christian Rohde, Sascha Tierling, Tomasz P. Jurkowski, Christoph Bock, Diana Santacruz, Sergey Ragozin, Richard Reinhardt, Marco Groth, Jörn Walter, & Albert Jeltsch

**Supplemental Text S5**. Methylation data of amplicons overlapping with SUZ12 binding regions.
