# Supplementary material for: DNA Methylation Analysis of Chromosome 21 Gene Promoters at Single Base Pair and Single Allele Resolution
Source: PLoS Genet. 2009 Mar 27;5(3):e1000438. doi: 10.1371/journal.pgen.1000438 (PMC2653639; doi:10.1371/journal.pgen.1000438)
Supplement: Text S6 — Allele-specific methylation. (0.06 MB DOC) [file pgen.1000438.s006.doc]

**DNA methylation analysis of chromosome 21 gene promoters at single base pair and single allele resolution**

Yingying Zhang, Christian Rohde, Sascha Tierling, Tomasz P. Jurkowski, Christoph Bock, Diana Santacruz, Sergey Ragozin, Richard Reinhardt, Marco Groth, Jörn Walter, & Albert Jeltsch

**Suppl. Text 6:** Allele-specific methylation

Allele-specific methylation of amplicon 197_intern from gene DSCR3 in leukocytes. The A/C SNP (dbsnp, rs764155) and C/T SNP (dbsnp rs764154) were identified. The positions of all SNP sites are indicated by arrow. Each row corresponds to one clone of bisulfite PCR products. Each column corresponds to one CpG site in the studied region. The color codes indicates the different methylation states of each CpG site. As reported before [16], the C-allele is unmethylated in leukocytes while the A-allele can be methylated or unmethylated.

Allele-specific methylation has been detected for the CBR1 (amplicon 176_1 and 176_2), C21orf81 (amplicon 23_1 and 23_2) and DSCR3 (amplicon 197_intern) genes in leukocytes. In the following table the corresponding results obtained in other tissues are compiled.

Abbreviations: del: deletion, m: methylated, unm: unmethylated
